# Immunomodulatory Role of an Ayurvedic Formulation on Imbalanced Immunometabolics during Inflammatory Responses of Obesity and Prediabetic Disease

**DOI:** 10.1155/2013/795072

**Published:** 2013-11-03

**Authors:** Kamiya Tikoo, Shashank Misra, Kanury V. S. Rao, Parul Tripathi, Sachin Sharma

**Affiliations:** Immunology Group, International Centre for Genetic Engineering and Biotechnology, Aruna Asaf Ali Marg, New Delhi 110067, India

## Abstract

Kal-1 is a polyherbal decoction of seven different natural ingredients, traditionally used in controlling sugar levels, inflammatory conditions particularly regulating metabolic and immunoinflammatory balance which are the major factors involved in obesity and related diseases. In the present study, we aimed to investigate the effect of Kal-1 (an abbreviation derived from the procuring source) on diet-induced obesity and type II diabetes using C57BL/6J mice as a model. The present study was performed with two experimental groups involving obese and prediabetic mice as study animals. In one, the mice were fed on high-fat with increased sucrose diet, and different amounts (5, 20, and 75 **μ**L) of Kal-1 were administered with monitoring of disease progression over a period of 21 weeks whereas in the second group the mice were first put on the same diet for 21 weeks and then treated with the same amounts of Kal-1. A significant reduction in body weight, fat pads, fasting blood glucose levels, insulin levels, biochemical parameters, immunological parameters, and an array of pro- and anticytokines was observed in obese and diabetic mice plus Kal-1 than control (lean) mice fed on normal diet. In conclusion, Kal-1 has immunomodulatory potential for diet-induced obesity and associated metabolic disorders.

## 1. Introduction

Globally, around 1.5 billion of the world's population are obese due to energy imbalance between calories consumed and calories expended [1]. Obesity is an inflammatory state particularly affecting the endocrine tissues mainly adipose tissues and is referred to as subclinical chronic mild inflammation, which is distinctive of clinical classic acute inflammation [2]. Fat tissue comprises a number of cell types primarily adipocytes, vascular endothelial cells, fibroblasts, and macrophages. Adipose is largely found in areas enriched for loose connective tissue. The urge to better understand the adipose architecture has provided answers to a lot of intriguing questions. The inflammation embarks on adipocytes, which are major endocrine cells that have specialization in lipid storage, and with the expansion of adipocytes mass (both hypertrophy and hyperplasia), inflammation increases. The chronic low-grade inflammation activates the innate immune system that subsequently leads to insulin resistance and further leads to type II diabetes mellitus. The mechanism and association of complete molecular and cellular inflammation between obesity and insulin resistance are just beginning to be revealed [3]. 

An important source of expansion or accumulation of fat in adipose tissue is consumption of high-fat high-sugar diet (HFHSD). Fat accumulation is closely associated with qualitative changes in lipoproteins (low- and high-density), cholesterol, and triglycerides. It is also suggested that free fatty acid production is increased during adipocyte mass expansion in obese state, and it is being implicated that it could be playing an important role in blocking the insulin signal transduction [4]. 

Adipose tissue is of two different types: the white (WAT) and brown (BAT) adipose tissues; both can be clearly distinguished at morphological and functional level. BAT is known for heat production by thermogenesis, whereas WAT is considered as important endocrine tissue and contributes to the pathogenesis of insulin resistance and regulation of metabolic inflammation [5]. WAT (subcutaneous and epididymal) is a known site for storing calories as triglycerides and main site of inflammation related to obesity [6]. Along with inflammatory modulators such as leptin, resistin, and adiponectin, a number of pro- (IL-1*β*, IL-6, IL-10, and TNF-*α*) and anti-inflammatory cytokines (IL-4 and IL-10) are also secreted by WAT. These adipokines dynamically affect metabolism as their production is considered to be regulated by nutritional state [7]. Anomalous production of mentioned adipocytokines and activation of inflammatory signaling pathways, namely, Jun N-terminal kinase (JNK) and inhibitor of NF-*κ*B kinase (IKK), are closely associated with chronic low-grade inflammation [8].

Though both WAT and BAT have unique specific roles in the body, WAT depending upon the location in the body can serve different functions [9]. The latter indicates that the variations in fat distribution in humans are correlated with metabolic disorders. In fact, now it is well appreciated that differentiated brown and white adipocytes have significant transcriptional, secretory, and morphological differences [10].

It is clear that the myriad of roles that adipose tissue plays in the body, together with an increasing relevance of understanding adipose tissue as it relates to obesity, calls for a need and importance of better understanding this tissue. A recent study done in mice using known stem cell surface markers has shown the importance of a set of markers which could be useful in enriching cells in fat tissue likely to be a white adipocyte precursor population [11]. However, there still remains lack of useful unique markers of white adipocyte precursors or adipocytes available for fat research [12]. These observations not only amplify the importance of understanding the histoarchitectural features of adipose tissue which can be clearly used as one of the significant markers underlying obesity. 

In modern era of pharmaceutical, most of the antiobesity and antidiabetic drugs have been found to be inconsistently effective and also have their associated side effects. As an alternative form of medicine, herbal or ayurvedic (a typical antique and religious type of medicine predominantly practiced in Asia) formulations are now being increasingly considered worldwide because of their utmost least toxic nature and side effects compared to synthetic drugs. These herbal formulations are well known to treat metabolic disorders including obesity and diabetes. For instance, Shao et al. [13] described the role of curcumin as an antiobesity and diabetes herbal medicine in an organized manner. Still, there are very limited systematic studies on the effect of herbal formulations on metabolic immunobalance in obese and diabetic individuals. 

This present study was conducted to test one such formulation, Kal-1, a polyherbal decoction of seven different natural ingredients (see Supplementary Table 1 in Supplementary Materials available online at http://dx.doi.org/10.1155/2013/795072), for its effect on the metabolic and immunoinflammatory balance in mouse model of diet-induced obesity and diabetes. Although Kal-1 is a proprietary product of Kerala Ayurveda, Kerala, India, the procuring source has authorized both (1) the use of Kal-1 name in the paper and (2) the listing of specific ingredients of Kal-1 in Supplementary Table 1. We evaluated the efficacy of Kal-1 as antiobesity and antidiabetic agent, in addition to its utility in controlling low-grade systemic inflammation and the overall energy equilibrium. We report here that, in addition to ameliorating the symptoms both of obesity and diabetes, Kal-1 administration also restored the normal balance of pro- versus anti-inflammatory cytokines, thereby skewing the immune response to more of anti-inflammatory type. Importantly, this activity was evident in regimens that probed possible potential value of Kal-1 to be explored further for supplementing it from the nutrient or food perspective to control imbalanced immune responses and resulting metabolic disease entities.

## 2. Materials and Methods

### 2.1. Preparation of Kal-1

Kal-1 formulation is essentially a concoction of seven different ingredients implicated to play a protective role in inflammation. It is prepared using a methodology prescribed by the ancient ayurvedic texts. Briefly, the different ingredients are washed, cleaned, dried, and sieved to get a coarse powder. The latter is then steamed and boiled till the starting volume reduces to one-eighth. This is followed by filtration and boiling till the decoction reaches one-fourth of the volume from the 1st filtrate (Supplementary Figure 9). The detailed protocol belongs to Kerala Ayurveda whose proprietary product is Kal-1. The resulting filtrate is then used as Kal-1, which is a dark brown liquid with a peculiar rotten leaf-like smell. Kal-1 dose to be administered in mice was calculated using human to mice dose conversion formula as described elsewhere [14].

### 2.2. Animals, Diets, and Experimental Setup

All animal studies were carried out at BIONEEDS Laboratory Animals & Preclinical Services, Bangalore, India, and approved by institutional animal ethics committee (IAEC). All experimental protocols were done as per applicable national and international guidelines. BIONEEDS is approved by committee for the purpose of control and supervision of experiments on animals (CPCSEA), Ministry of Forests and Environments, Government of India. Briefly, 3-4-week-old male C57BL/6J mice (7–9 gm) were housed (3–5 animals per cage) under standard conditions. All animals were initially put on two different diets (normal diet (LFD, D12492) containing 10% kcal fat and high fat with increased sugar diet (HFHSD, D03062301) containing 60% kcal fat in pellet forms, procured from Research Diet, NJ, USA). The first two weeks without the formulation were considered as “acclimatization phase” wherein mice were given their respective diets only before starting the formulation. The administration of Kal-1 was done in two different disease rescue (up to 21 weeks) and treatment (up to 30 weeks) experiments in which mice were divided into five groups—LFD control group, HFHSD control group, and three different amounts of Kal-1 (5, 20, and 75 *μ*L) supplemented with high-fat high-sugar diet (HFHSD + Kal-1) test groups. In rescue experiments, all three amounts of Kal-1 were administered for next 19 weeks after acclimatization period, whereas Kal-1 with the same amounts were administered only for 8 weeks after 22 weeks in treatment experiments. The feed intake was monitored daily, which included residual feed quantification as well, and body weights were recorded twice a week.

### 2.3. Tissues Isolation and Blood Collection

At an interval of three weeks, that is, weeks 3, 6, 9, 12, 15, 18, and 21 over the entire experimental period, mice were kept on fasting for a period of 5-6 hours prior to blood collection and then anesthetized with ether in rescue experiment, whereas the same procedure was followed only at weeks 26 and 30 in treatment experiment. White adipose tissues (epididymal and subcutaneous) fat depots were removed carefully in both the cases at mentioned time points, thoroughly rinsed with phosphate buffer saline, and weighed.

Blood was collected from retroorbital sinus for serum separation. Blood glucose levels were measured by using a glucometer (Roche Diagnostics GmbH, Germany) at the above-mentioned time periods.

### 2.4. Biochemical Analysis

The serum concentrations of low-density lipids (LDL), high-density lipids (HDL), total cholesterol, and triglycerides were assayed enzymatically by using an automatic analyzer (ERBA, automated random access clinical chemistry analyzer, EM260, Mannheim, Germany) with their respective kits. The serum insulin levels were measured using commercially available ELISA kit (ALPCO ultrasensitive mouse insulin kit, Salem, NH, USA) for mouse. Leptin and resistin levels were measured using commercially available radioimmunoassay kits (Quantikine, mouse leptin and mouse resistin, immunoassay, R&D, Minneapolis, MN, USA) and high-molecular-weight adiponectin (ALPCO, adiponectin mouse total, HMW, Salem, NH, USA).

### 2.5. Cytokine Measurement

An array of seven cytokines (pro- and anti-inflammatory) was measured in serum of the experimental groups. Briefly, sera from the blood were used to detect the following cytokines, namely, IL-1*α*, IL-1*β*, IL-4, IL-6, IL-10, TNF-*α*, and MCP-1, using the Luminex system (Liquichip 200, Luminex xMAP Technology, Valencia, CA, USA) as per the manufacturer's protocols. Kits for multiplex analysis were obtained from Millipore, Billerica, MA, USA. All samples were run in triplicates.

### 2.6. Statistics

To check the significant difference between LF and HF control groups, we performed Student's *t*-tests whereas one-way ANOVA followed by Tukey's multiple comparison test was used to compare between control groups (LF and HFHS) and test groups (HFHS supplemented with different amounts of Kal-1 doses). *P* value less than 0.05 was considered to be statistically significant. All the data was expressed as mean ± SEM (*n* = 5, each group). Statistical analysis was performed using GraphPad Prism software (GraphPad Software, San Diego, CA, USA).

## 3. Results

### 3.1. Simultaneous Kal-1 Administration Mitigates the Effects of HFHSD on Systemic Inflammation and Metabolic Dysfunction in Mice

Here, we evaluated the effect of LFD, HFHSD, and Kal-1 supplemented HFHSD group in C57BL/6J mouse model up to 21 weeks. We monitored body weights, metabolic biochemical parameters, and immunological readouts like hormones and cytokines.

To assess the potential effects of KAL-1 on body weight regulation, we observed body weights of animals fed on LFD and HFHSD for a period of twenty-one weeks (a period including fifteen days of acclimatization). Wherein, we screened several amounts of Kal-1 ranging from very low (0.04 *μ*L) to high amounts (300 *μ*L). A total of 275 mice were grouped (5 mice/group) into eleven different groups, namely, LF control, HFHS control, and HFHS supplemented with nine different test amounts Kal-1. A clear-cut dose-dependent effect of Kal-1 was observed over the entire experimental phase (week 3 to week 21) at all amounts of Kal-1. However, this trend excluded the two higher amounts of 150 and 300 *μ*L (that could be potentially toxic). Body weights at Kal-1 amounts of 5, 20, 38, and 75 *μ*L were observed to be closely comparable to LF control group (Supplementary Figure 1). Therefore, for all further experiments, we mainly focused on three Kal-1 amounts of 5, 20, and 75 *μ*L. 

**Figure 1:**
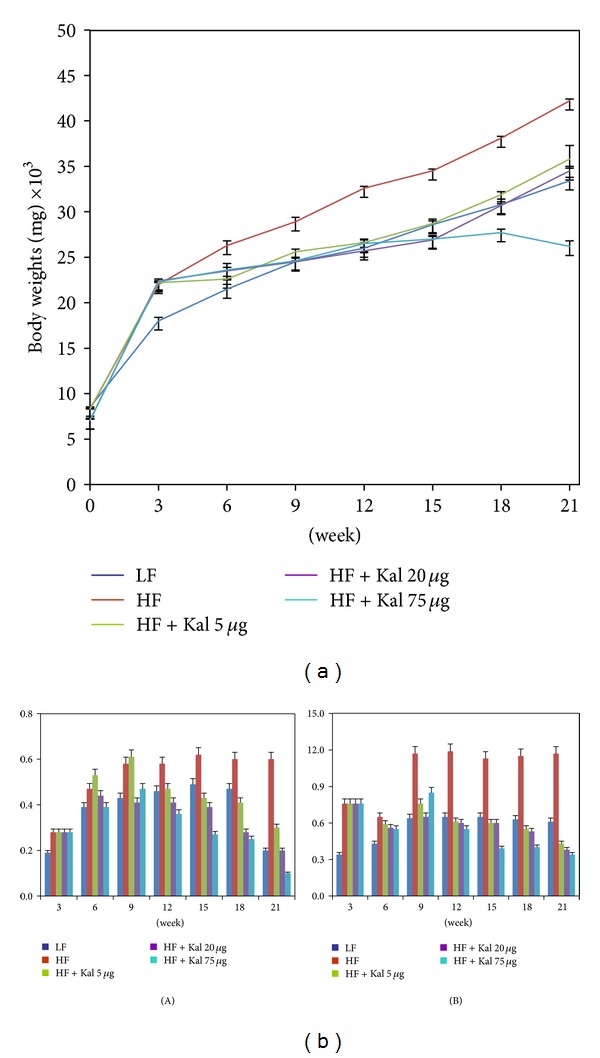
Body and tissue weights in normal diet fed control were higher than high-fat high-sugar diet fed control, and Kal-1 doses rescue mice fed on high-fat high-sugar diets from being obese. (a) Weekwise effect of Kal-1 on body weights in high-fat high-sugar fed mice. (b) Effect of Kal-1 on tissue weights in high-fat high-sugar fed mice at week 21. (A) Epididymal fat; (B) subcutaneous fat. All doses (5, 20, and 75 *μ*L) of Kal-1 were supplemented along with HFHSD. LF: low-fat control, HF: high-fat high-sugar control. All the values represent mean ± SEM from five animals.

Additionally, a significant difference in mean body weights of LF and HFHS control groups was observed (8.8 gm or 26.3%, *P* < 0.0005; Figure 1(a)); HFHSD-fed animals were heavier than LFD-fed animals at week 21. The closer group to LF control group was HFHS + 20 *μ*L Kal-1 with the difference of 1.1 gm or 3%. A difference in body weights of HFHS + 5 *μ*L Kal-1 and LF control group was 2.4 gm or 7.1%. Though, HFHS + 75 *μ*L Kal-1 treatment group showed a different profile (lower than LF control group) with differences in body weights being 7.2 gm or 21.5% (Figure 1(a)). However, all the differences among experimental and control groups were found to be statistically nonsignificant (*P* > 0.05) when analyzed by ANOVA followed by Tukey's multiple comparison test.

Further, in order to ensure that any changes or effects observed were true effects of Kal-1, gavage control alone was also put (wherein the mice were administered same volume of distilled water). There was no difference observed in the weight of the animals between the control groups and gavage control group (Supplementary Figure 2).

Furthermore, similar effect of Kal-1 was also observed on the weights of fat pads, that is, WAT. At week 21, the mean of relative weights of WAT, namely, epididymal and subcutaneous fat depots, was significantly higher in HFHS control animals than LF control animals (4 gm and 5.6 gm, resp.). Similarly, epididymal fat pads on analysis using one-way ANOVA followed by Tukey's multiple comparison test for experimental (HFHS + KAL-1 20 *μ*L and 75 *μ*L treated animals) and control group (LFD) were found to be different but not significant. Differences among experimental (HFHS + KAL-1 5 *μ*L, 20 *μ*L, and 75 *μ*L treated animals) and control groups (LFD) on analysis using one-way ANOVA followed by Tukey's multiple comparison test for weight of subcutaneous fat pads were also found to be nonsignificant (Figure 1(b)).

### 3.2. Amount of Feed Taken Does Not Affect the Weight of Animals as Confirmed by Pair Fed Experiments

Since gain in body weight was significantly higher in mice receiving HFHSD as compared to the group being fed on LFD and the HFHSD + Kal-1 treated group was almost comparable with LFD control group, we measured the feed intake and body weights up to 21 weeks in all the groups including pair-fed group to demonstrate that the difference between LFD and HFHSD control groups was due to high carbohydrate with increased sucrose content in HFHSD, not due to more eating of HFHSD (Supplementary Figures 3 and 4). Furthermore, to verify the effect of Kal-1 on body weights of mice, we monitored the *ad libitum *feed intake in HFHSD fed control mice and restricted the amount of feed to HFHSD + pair-fed control and HFHSD + pair-fed + Kal-1 test groups. Supplementary Figure 4 shows that body weights of HFHSD + pair-fed control and Kal-1 supplemented test groups gained weight (47.0 and 35.1 gm, resp.) almost similar to HFHSD control group and LFD control groups (47.9 and 35.7 gm, resp.) at week 21. These findings indicate that HFHSD diet promoted increase in animal body mass, and KAL-1 is effective in reducing body weight gain.

Furthermore, there was no observable change in the core body temperature in either control or Kal-1 supplemented test group. Rectal temperature of all the animals from pair-feeding experiment was also recorded for all four groups from week 15 to week 21 and it was ranging from 94.2°F to 98.2°F (Supplementary Figure 5), which was again normal.

### 3.3. Kal-1 Rectifies the Metabolic Imbalance in HFHSD Fed Mice

#### 3.3.1. Fasting Blood Glucose and Insulin Profiles

To determine that high-calorie diet results in a shift in immunobalance which leads to symptoms towards development of diabetes, blood glucose levels were also recorded. This was done after 5-6 hours of fasting for all 275 animals which were further grouped (11 groups) in the same manner as mentioned earlier (Supplementary Figure 6).

**Figure 2:**
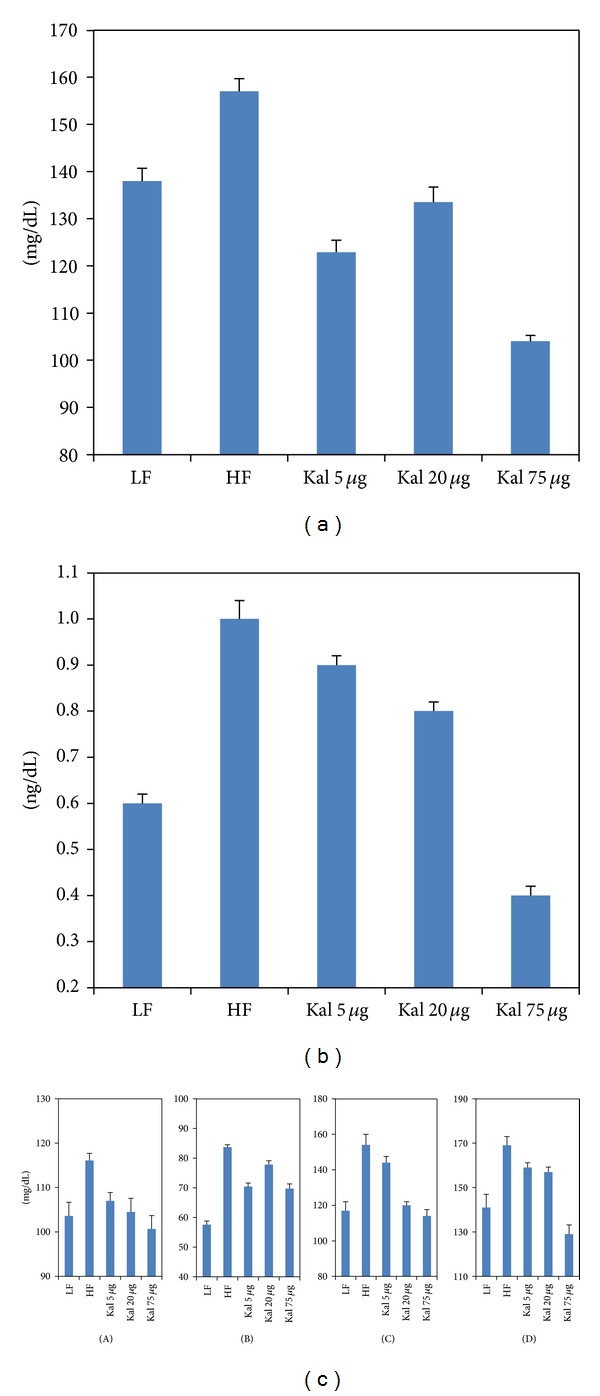
Kal-1 rectifies the metabolic imbalance in mice fed on high-fat high-sugar diets. (a) Effect of Kal-1 on fasting blood glucose levels in high-fat high-sugar fed diet at week 21. (b) Effect of Kal-1 on fasting insulin levels in high-fat high-sugar fed diet at week 21. (c) Effect of Kal-1 on various biochemical parameters (fasting) in high-fat high-sugar fed diet at week 15. (A) HDL. (B) LDL. (C) Cholesterol. (D) Triglycerides. All doses (5, 20, and 75 *μ*L) of Kal-1 were supplemented along with HFHSD. LF: low-fat control, HF: high-fat high-sugar control. All the values represent mean ± SEM from five animals.

In comparison to LFD fed animals, blood glucose levels were significantly elevated (*P* < 0.005) in HFHSD fed animals. Differences among experimental (HFHS + KAL-1 5 *μ*L and 75 *μ*L treated animals) and control group (LFD) were found to be statistically significant (*P* < 0.05) whereas HFHS + KAL-1 20 *μ*L test animals were found to be nonsignificant though comparable on analysis using one-way ANOVA followed by Tukey's multiple comparison test (Figure 2(a)).

Fasting serum insulin levels were also measured for LF, HFHS, HFHS + Kal-1 5 *μ*L, HFHS + Kal-1 20 *μ*L, and HFHS + Kal-1 75 *μ*L test groups. At week 21, serum insulin levels were 0.2 ng/dL, 0.3 ng/dL, and 0.4 ng/dL higher in HFHS + Kal-1 20 *μ*L, HFHS + Kal-1 5 *μ*L and HFHS groups, respectively, than LF animals. On analysis of differences among HFHS and HFHS + Kal-1 5 *μ*L, HFHS + Kal-1 20 *μ*L, and HFHS + Kal-1 75 *μ*L groups using one-way ANOVA followed by Tukey's multiple comparison test, and it was found to be significant only for HFHS and HFHS + Kal-1 75 *μ*L groups (*P* < 0.05).

#### 3.3.2. Effect on HDL, LDL, Cholesterol, and Triglycerides Levels

At week 15, nonsignificant elevated levels (12%, 45.3%, 31.6%, and 19.8%) of fasting serum in HFHS control animals were noticed for HDL, LDL, cholesterol, and triglycerides, respectively, as compared to LF group. Similar to body weight and insulin profile, dose-dependent effects of Kal-1 were seen in HDL, cholesterol, triglycerides, and LDL levels. However, differences among LF and HFHS + Kal-1 5 *μ*L, HFHS + Kal-1 20 *μ*L, and HFHS + Kal-1 75 *μ*L groups were found to be significant with HFHS + Kal-1 5 *μ*L for cholesterol whereas both groups HFHS + Kal-1 5 *μ*L and HFHS + Kal-1 20 *μ*L were found to be significant (*P* < 0.05) for LDL levels and nonsignificant for HDL and triglycerides levels on analysis using one-way ANOVA followed by Tukey's multiple comparison test.

### 3.4. Kal-1 Corrects Immunological Readouts in HFHSD Fed Mice

HFHSD altered all the immunological readouts, namely, hormones and cytokines pattern in HFHSD group in comparison to LFD group. These altered patterns came back to the normal when Kal-1 was administered in the similar groups of HFHSD, and these parameters were tracked over the same time phase as done earlier for body weights and biochemical readouts.

### 3.5. Hormone and Cytokine Production is Affected by HFHSD Intake and Kal-1 Administration

**Figure 3:**
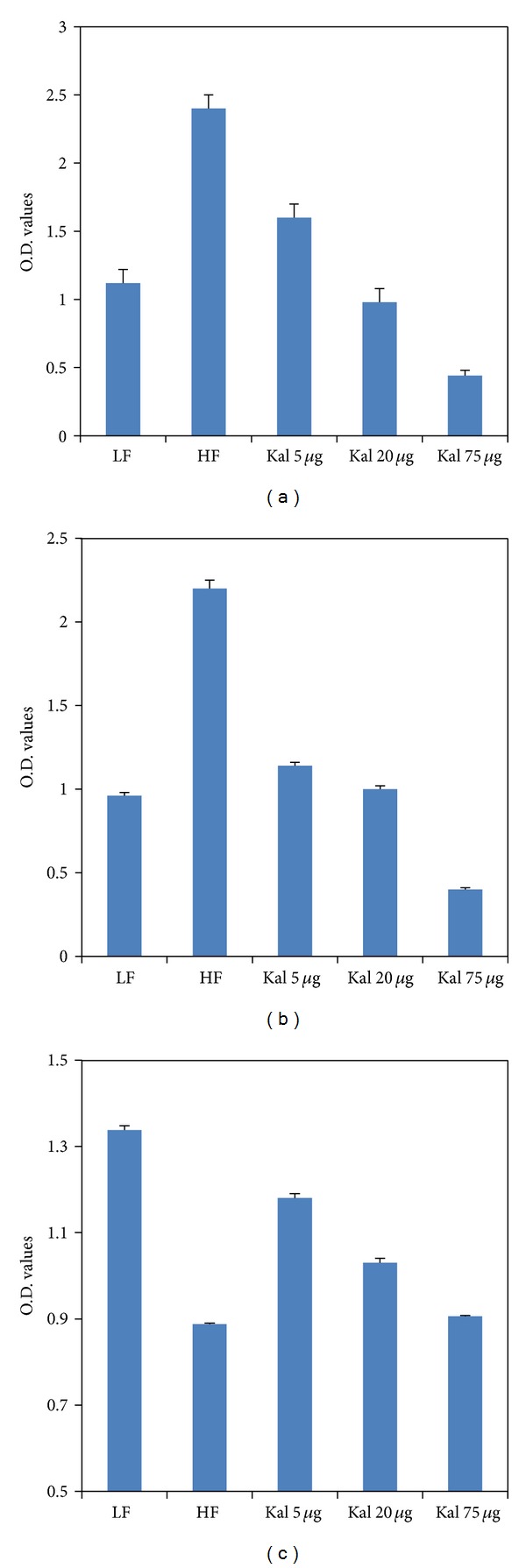
Kal-1 rectifies the hormonal imbalance in mice fed on high-fat high-sugar diets. (a) Effect of Kal-1 on resistin levels in high-fat high-sugar fed mice at week 15. (b) Effect of Kal-1 on leptin levels in high-fat high-sugar fed mice at week 15. (c) Effect of Kal-1 on high-molecular-weight adiponectin levels in high-fat high-sugar fed mice at week 15. All doses (5, 20, and 75 *μ*L) of Kal-1 were supplemented along with HFHSD. LF: low fat control, HF: high-fat high-sugar control. All the values represent mean ± SEM from five animals.

We noticed dose-dependent effect of Kal-1 for all three hormones, namely, resistin, leptin, and HMW adiponectin at week 15. The differences between LFD and HFHSD control groups were 50% (*P* < 0.005), 56% (*P* < 0.0005), and 38% (*P* < 0.0001) for resistin, leptin, and HMW adiponectin, respectively, which were statistically significant (Figures 3(a), 3(b), and 3(c)). Regulation for both leptin and resistin was exhibited at Kal-1 dose of 20 *μ*L which was in concordance with other previous observations. However, comparison between LF and HFHS + Kal-1 5 *μ*L; HFHS + Kal-1 20 *μ*L and HFHS + Kal-1 75 *μ*L groups was found to be significant with HFHS + Kal-1 75 *μ*L (*P* < 0.05) only on analysis using one-way ANOVA followed by Tukey's multiple comparison test for leptin. Differences among experimental (HFHS + Kal-1 5 *μ*L, HFHS + Kal-1 20 *μ*L, and HFHS + Kal-1 75 *μ*L groups) and control groups (HFD) on analysis using one-way ANOVA followed by Tukey's multiple comparison test were found to be statistically significant (*P* < 0.05) with HFHS + Kal-1 5 *μ*L and HFHS + Kal-1 20 *μ*L for adiponectin. 

Two panels of cytokines, namely, pro- (IL-1*α*, IL-1*β*, IL-6, MCP-1, and TNF-*α*) and anti-inflammatory (IL-4 and IL-10), were analyzed in serum at week 15. Statistically significant differences were observed in LFD and HFHSD groups (33 pg/mL, *P* < 0.0001 and 38 pg/mL, *P* < 0.0001) for IL-4 and IL-10 concentrations, respectively. These results suggest that increased body weight and related metabolic disorders due to HFHSD also affected the concentrations of anti-inflammatory cytokines.

Similar to IL-4 and IL-10, *t*-test showed that all studied proinflammatory cytokines were significantly different between LFD and HFHSD fed animals. The highly significant difference in values ranged between 57 pg/mL (*P* < 0.00001), 161 pg/mL (*P* < 0.00001), 112 pg/mL (*P* < 0.00001), 67 pg/mL (*P* < 0.000008), and 226 pg/mL (*P* < 0.000005), respectively for IL-1*α*, IL-1*β*, IL-6, MCP-1, and TNF-*α*.

Differences among experimental group (HFHS + Kal-1 5 *μ*L, HFHS + Kal-1 20 *μ*L, and HFHS + Kal-1 75 *μ*L groups) and control group (LFD) on analysis using one-way ANOVA followed by Tukey's multiple comparison test were found to be statistically significant (*P* < 0.05) for all with IL-4 and nonsignificant for HFHS + Kal-1 75 *μ*L group with IL-10. Comparison between experimental (HFHS + Kal-1 5 *μ*L, HFHS + Kal-1 20 *μ*L, and HFHS + Kal-1 75 *μ*L groups) and control group (LFD) for pro- (IL-1*α*, IL-1*β*, IL-6, MCP-1, and TNF-*α*) inflammatory cytokines was found to be statistically significant (*P* < 0.05) except for IL-1*α* for which HFHS + Kal-1 20 *μ*L group was nonsignificant on analysis using one-way ANOVA followed by Tukey's multiple comparison test.

Thus, both of the readouts, namely, hormones and cytokines concentrations, showed marked levels of correction in Kal-1 supplemented group.

### 3.6. Kal-1 Treatment Restores the Inflammatory Balance in Mice Fed on HFHSD

We also monitored the effect of Kal-1 after 21 weeks of obesity induction by feeding the mice on HFHSD, which was the reverse of what we did earlier. Here also we observed similar trends in correction for body weights, fasting blood glucose, blood biochemistry, serum hormones, and cytokines in the same manner as described earlier with the same number of mice per group.

Kal-1 at the three above-mentioned amounts (5, 20, and 75 *μ*L) was then put forth to be administered for next 8 weeks in the HFHSD group. We observed that 8-week short-term dietary treatment of Kal-1 at 75 *μ*L significantly reduced (*P* < 0.05) the body weight of HFHSD fed animals instead of HFHSD + Kal-1 at 20 *μ*L test animals (as in rescue experiments) on comparison among experimental (HFHS + Kal-1 5 *μ*L, HFHS + Kal-1 20 *μ*L, and HFHS + Kal-1 75 *μ*L group) and control groups (HFD) using one-way ANOVA analysis followed by Tukey's multiple comparison test (Figure 5(b)), though the effect of Kal-1 was again dose dependent as seen earlier (Figure 5(a)(A, B)). 

### 3.7. Treatment with Kal-1 Modulates Blood Glucose and Serum Insulin Levels in Obesity-Induced Mice by HFHSD

Fasting blood glucose and serum insulin levels were measured at week 30 in experimental mice to assess the effect of treatment with Kal-1 on these aspects. Fasting blood glucose value of HFHSD fed mice was significantly higher (25 mg/dL or 17%, *P* < 0.005) than LFD fed mice at week 30. The mean concentrations of fasting blood glucose in the Kal-1 75 *μ*L treated mice were significantly less (22 mg/dL, 15% *P* < 0.05) than in mice fed on HFHSD. The rest two amounts of 5 *μ*L and 20 *μ*L of Kal-1 treated animals were found to be nonsignificant on analysis using one-way ANOVA followed by Tukey's multiple comparison test (Figure 5(b)). Moreover, the fasting serum insulin levels were increased >2-fold in HFHS grouped mice than LF grouped mice. Differences among all experimental (HFHS + Kal-1 5 *μ*L, HFHS + Kal-1 20 *μ*L, and HFHS + Kal-1 75 *μ*L groups) and control groups (HFD) on analysis using one-way ANOVA followed by Tukey's multiple comparison test were found to be statistically significant (*P* < 0.05). The levels of HFHS + Kal-1 5 and 20 *μ*L test group were also comparable with LF control group but not as closer as those HFHS + Kal-1 75 *μ*L test group (Figure 5(c)).

### 3.8. Functional Relevance of Dietary Treatment of Kal-1 on Serum Biochemistry

Following the same approach, serum biochemistry of individual mice in all the five groups (control groups and Kal-1 treated groups) were also examined. All parameters like serum HDL, LDL, cholesterol, and triglycerides were distinguishable between LF and HFHS control groups, although the differences in only HDL and cholesterol levels were statistically significant. The values for HDL and cholesterol of HFHS + Kal-1 75 *μ*L test group were comparable (6 mg/dL or 7.5% and 17 mg/dL or 15.7%, resp.) with LF control group. However, the same was statistically significant (*P* < 0.05) only for cholesterol on analysis using one-way ANOVA followed by Tukey's multiple comparison test. In contrast, values of LDL and triglycerides for HFHS + Kal-1 75 *μ*L test group were found to be little less (1 mg/dL or 2.3% and 3 mg/dL or 2.6%) than LF control group and nonsignificant on analysis using one-way ANOVA followed by Tukey's multiple comparison test (Figures 6(a), 6(b), 6(c), and 6(d)). 

### 3.9. Antiobesity Effect of Kal-1 as Assessed through Measurement of Hormones and Cytokines Secreted during Disease State

To further examine the treatment effect of Kal-1 on pro- and anti-inflammatory parameters, a set of two hormones and seven cytokines was analyzed in the serum of LF and HFHS controls and HFHSD fed mice supplemented with Kal-1. For serum leptin and HMW adiponectin levels, a significant alteration was detected in LF and HFHS control groups (*P* < 0.02 and *P* < 0.05, resp.) at week 30. And differences among experimental (HFHS + Kal-1 5 *μ*L, HFHS + Kal-1 20 *μ*L, and HFHS + Kal-1 75 *μ*L groups) and control groups (HFD) on analysis using one-way ANOVA followed by Tukey's multiple comparison test were found to be statistically significant (*P* < 0.05) with HFHS + KAL-1 20 *μ*L and 75 *μ*L for leptin only and nonsignificant with HFHS + KAL-1 5 *μ*L treated animals for both leptin and adiponectin (Figures 7(a) and 7(b)).

Figure 8(a) revealed that serum levels of LF control mice were 40% (*P* < 0.01) and 47% (0.005) higher than those of HFHS control mice in anti-inflammatory cytokines, IL-4 and IL-10, respectively. On the other hand, 72% and 65% statistically significant decrease (*P* < 0.05) in the levels of both cytokines—IL-4 and IL-10, respectively—were observed for HFHS + Kal-1 75 *μ*L group than LF control group. However, the levels of both cytokines in all test groups (HFHS + Kal-1 5 *μ*L, 20 *μ*L, and 75 *μ*L) were found to be nonsignificantly lower than HFHS control group at week 30. The analysis was done using one-way ANOVA followed by Tukey's multiple comparison test, whereas at week 30, HFHSD fed animals showed significant increase in the pro-inflammatory cytokines levels (Figure 8(b)), when compared with LFD fed animals (*P* < 0.005 for all except MCP-1), while Kal-1 at 75 *μ*L resulted in significant reductions of 259 pg/mL, 198 pg/mL, 123 pg/mL, 88 pg/mL, and 382 pg/mL on the IL-1*α*, IL-1*β*, IL-6, MCP-1, and TNF-*α* levels, respectively, in the serum of HFHSD fed animals (*P* < 0.05 for all except MCP-1 and IL-6) at week 30 on comparison among experimental (HFHS + Kal-1 5 *μ*L, HFHS + Kal-1 20 *μ*L, and HFHS + Kal-1 75 *μ*L groups) and control groups (HFD) on analysis using one-way ANOVA followed by Tukey's multiple comparison test. 

## 4. Discussion

The present study confirms immunoregulatory effect of Kal-1, an ayurvedic formulation suggestive of controlling obesity and diabetes. Kal-1 is basically a decoction of seven different ingredients (with synergistic properties) which we suggest (based on information provided by the procuring source) could be useful in regulating heightened or disturbed immune response especially during chronic low-grade inflammatory conditions, namely, obesity and diabetes. We tested the above formulation in well-established diet-induced mice models (C57BL/6J strain of mice) using skewing of immune response from a pro- to anti-inflammatory as one of the key elementary readouts. The effect of Kal-1 on body fat mass, adipose tissues (epididymal and subcutaneous) weights, blood biochemistry including blood glucose level and insulin profile, and adipocytokines were monitored on HFHSD-induced experimental mice.

A number of *in vivo* studies have shown the effect of low- and high-fat diets on body weights, blood glucose level, and inflammatory markers [15–18]. It is noteworthy that diets play an important role in inflammatory modulation; especially high-carbohydrate diet directly contributes to fat mass expansion in adipose tissues and then leads to inflammation and insulin resistance [19]. In the present study, it is observed that HFD with increased sucrose significantly elevates the body weights, blood glucose, and serum insulin levels in mice than LFD fed mice over the observation period. Moreover, preliminary screening study of body weights in mice fed on HFHSD with the different dose amounts of Kal-1 was also performed; consequently, Kal-1 dose-dependent reduction in body weights was also observed (Supplementary Figure 1). On the basis of body weights reduction profile, a single amount of Kal-1, that is, 20 *μ*L, was optimized. This was based on the observation that body weights of animals being fed on HFHSD along with this amount were equal to body weights of LF fed mice in rescue experiment (Figure 1(a)). Surprisingly, same type of observations were also noticed with Kal-1 in treatment study (Figure 5(a)(A)); however, 75 *μ*L, a higher amount of Kal-1, was the optimal dose (Figure 5(a)(B)). In the same manner, a decrease in the weights of epididymal and subcutaneous fat pads was also exemplified by Kal-1 (Figure 1(b)(A, B)).

With these observational facts, it may be speculated that Kal-1 is effective either at the level of regulating adipocyte hypertrophy, adipogenesis, or both. Second, alteration in the fatty acids present in HFHSD from monounsaturated fatty acid to saturated fatty acid due to Kal-1 is another possibility as circulating saturated fatty acids play a key role in obesity [20]. It must, however, be taken into account that HFHSD comprised almost equal amounts of both saturated and monounsaturated fatty acids (details not given).

Despite the fact that chronic low-grade inflammation is directly linked with the consumption of high-carbohydrate diets [21], sucrose is one of the important elements in HFD which is the leading cause of obesity, high blood sugar, and insulin resistance [22]. In this respect, expected higher levels of blood glucose and serum insulin levels were observed in obese mice control group (fed on HFHSD) than in lean mice control group (fed on LFD). Irrespective of the HFHS constituents in diet, significantly lower levels of blood glucose and serum insulin were observed in both test groups administered with Kal-1 20 and 75 *μ*L (Figures 2(a), 2(b), 5(b)(B), and 5(c)), which could not be explained.

Furthermore, one metabolically active hormone is resistin, which is secreted by adipocytes and may contribute to obesity, insulin resistance, and diabetes in mice. In parallel with the observations of Steppan and Lazar [23], here we also show that serum resistin levels of lean mice were reduced up to more than 2-fold compared to obese mice. In accordance with glucose and insulin profile, decreased levels of resistin were observed in obese mice exposed to Kal-1 and were comparable to those in lean mice (Figure 3(a)). Therefore, it can be speculated that Kal-1 exhibits similar effects on resistin levels as is observed for blood glucose and serum insulin levels [23, 24].

Further, leptin is an adipokine, also secreted by adipocytes, considered a key pro-inflammatory cytokine. In addition to regulating food intake and energy homeostasis, this bioactive molecule also plays a potent role in modulating the immune response and inflammatory processes. Leptin is present in serum in direct proportion to the amount of adipose tissue; therefore, sum of energy in adipose tissue reveals the level of leptin in serum; that is, the more the energy the more the production of leptin. Similar to previous explanation [25], amount of energy stored in adipocytes of obese mice fed with HFHSD was higher than that in mice fed with LFD as leptin levels were found to be significantly more in obese mice than lean mice in current study. In corroboration with previous studies, increased leptin production can be positively correlated with adipocyte hypertrophy and hyperplasia [26, 27]. In both experiments, the serum from mice on HFHSD supplemented with Kal-1 showed that leptin levels came back to the normal levels almost comparable with the levels seen in the LF diet group (Figures 3(a) and 7(a)). One possible explanation could be that Kal-1 stimulates and catalyses lipolysis and at the same time also regulates excess accumulation of fat cells in the body. 

Unlike leptin, adiponectin, an adipocyte-specific secretary protein, is well known for its anti-inflammatory action. Shklyaev et al. [28] reported that adiponectin with sustained peripheral expression can improve insulin sensitivity too. The hormone also contributes to the production of anti-inflammatory cytokines and suppresses the pro-inflammatory cytokines [29]. Serum adiponectin levels decrease with obesity or with increased adiposity, though the mechanism behind this reduction is still unclear. Similar to the above-mentioned fact, the adiponectin concentration shrinks with body weight reduction after the administration of Kal-1 in rescue and treatment studies in the present investigation (Figures 3(c) and 7(b)). Consequently, it can be hypothesized that Kal-1 might be a contributing factor for reduction in body weight. 

Production and regulation of adipocytokines from adipocytes have been shown to be completely based on dietary conditions as dietary fats are directly associated with obesity and related metabolic disorders like diabetes. LF diet accompanies decreased inflammatory markers whereas HFHS diet improved levels of pro-inflammatory cytokines [30]. In case of obesity and impaired glucose metabolism, chronic low-grade inflammation is considered to be a principal mechanism. The chronic inflammation can be only controlled by equilibrium between pro-inflammatory and anti-inflammatory cytokines. 

Out of a number of adipocytokines expressed in and secreted by adipocytes, namely, IL-1*α*, IL-1*β*, IL-6, TNF-*α*, and MCP-1 are considered as classical pro-inflammatory cytokines in chronic inflammatory responses. It has been implicated by earlier studies that these cytokines are involved in the low-grade inflammation, impaired glucose metabolism, and insulin resistance [31–35]. In accordance with previous studies, it is revealed in the present study that mentioned pro-inflammatory cytokines concentrations are higher in HFHSD fed animals in comparison to LFD fed animals. The serum levels of TNF-*α* and IL-6, key pro-inflammatory cytokines, are frequently increased in the obese state which is well in concurrence with the present study. TNF-*α* actively participates in the development of insulin resistance and IL-6 is linked with type II diabetes. IL-1*α* (cell-associated molecule) and IL-1*β* (secretary protein) are members of IL family and recognized as immunomodulatory proteins. Both are related with obesity while IL-1*β* is also linked with obesity-induced diabetes [6, 35]. It has been reported that one of the important pro-inflammatory cytokines is MCP-1 whose circulating levels were high in the obese mice model. Contrary to this, in our study, decreased serum concentrations of two important adipokines IL-4 and IL-10 were found in obese animals compared to lean animals. This finding is in support of the fact that these adipokines have long been considered as anti-inflammatory cytokines [36, 37].

**Figure 4:**
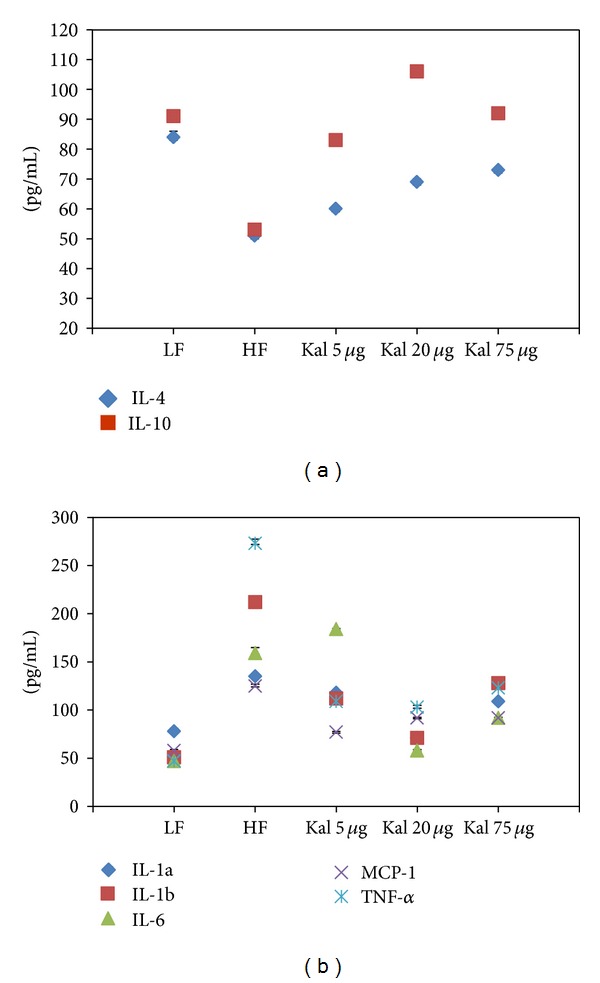
Kal-1 rectifies the inflammatory cytokines imbalance in mice fed on high-fat high-sugar diets. (a) Effect of Kal-1 on anti-inflammatory cytokines in high-fat high-sugar fed mice at week 15. (b) Effect of Kal-1 on proinflammatory cytokines in high-fat high-sugar fed mice at week 15. All doses (5, 20, and 75 *μ*L) of Kal-1 were supplemented along with HFHSD. LF: low-fat control, HF: high-fat high-sugar control. All the values represent mean ± SEM from five animals.

**Figure 5:**
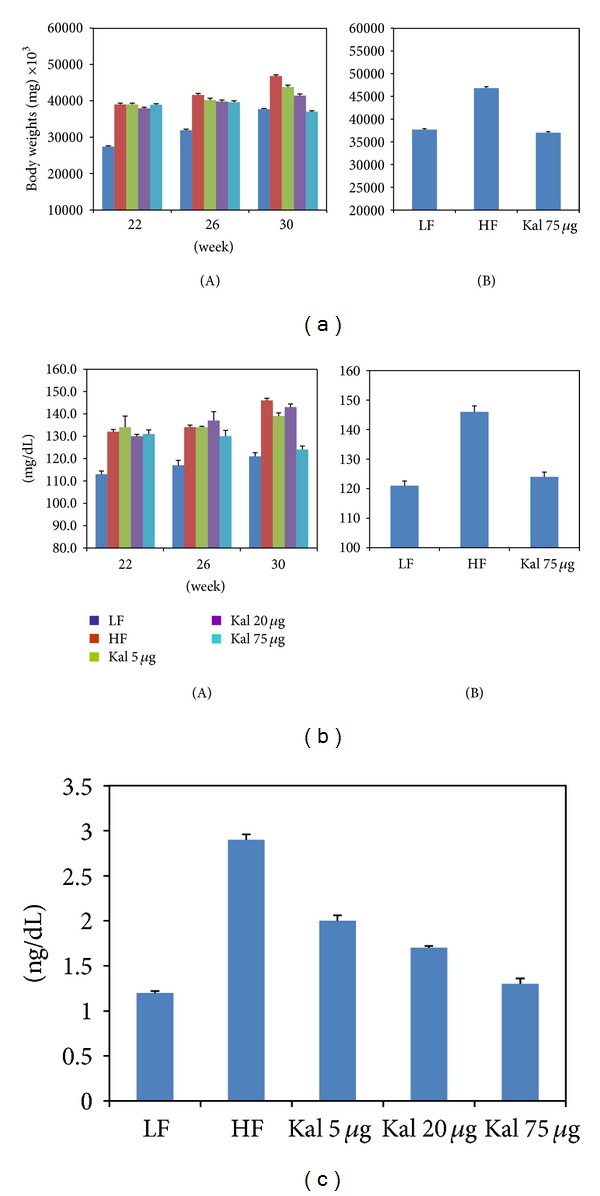
Body weights, fasting blood glucose, and insulin levels in high-fat high-sugar fed control were lower than normal diet fed control mice and Kal-1 treatment restored the body weights and blood glucose levels successfully. After obesity and diabetes induction period up to 21 weeks, Kal-1 treatment was started from week 22 to 30. (a)(A) Effect of Kal-1 treatment on body weights in high-fat high-sugar diet fed mice at weeks 22, 26, and 30. Treatment with all doses (5, 20, and 75 *μ*L) of Kal-1 was started only after 21 weeks (induction period) along with high-fat high-sugar diet. (B) Effect of Kal-1 treatment with optimum dose (75 *μ*L) on body weights in high-fat high-sugar diet fed mice only at week 30. (b)(A) Effect of Kal-1 treatment on fasting blood glucose levels in high-fat high-sugar diet fed mice at weeks 22, 26, and 30. Treatment with all doses (5, 20, and 75 *μ*L) of Kal-1 was started only after 21 weeks (induction period) along with high-fat high-sugar diet. (B) Effect of Kal-1 treatment with optimum dose (75 *μ*L) on fasting blood glucose in high-fat high-sugar diet fed mice only at week 30. (c) Effect of Kal-1 treatment on fasting insulin levels in high-fat high-sugar diet fed mice at week 30. Treatment with all doses (5, 20, and 75 *μ*L) of Kal-1 was started only after 21 weeks (induction period) along with high-fat high-sugar diet. LF: low-fat control, HF: high-fat high-sugar control. All the values represent mean ± SEM from five animals.

**Figure 6:**
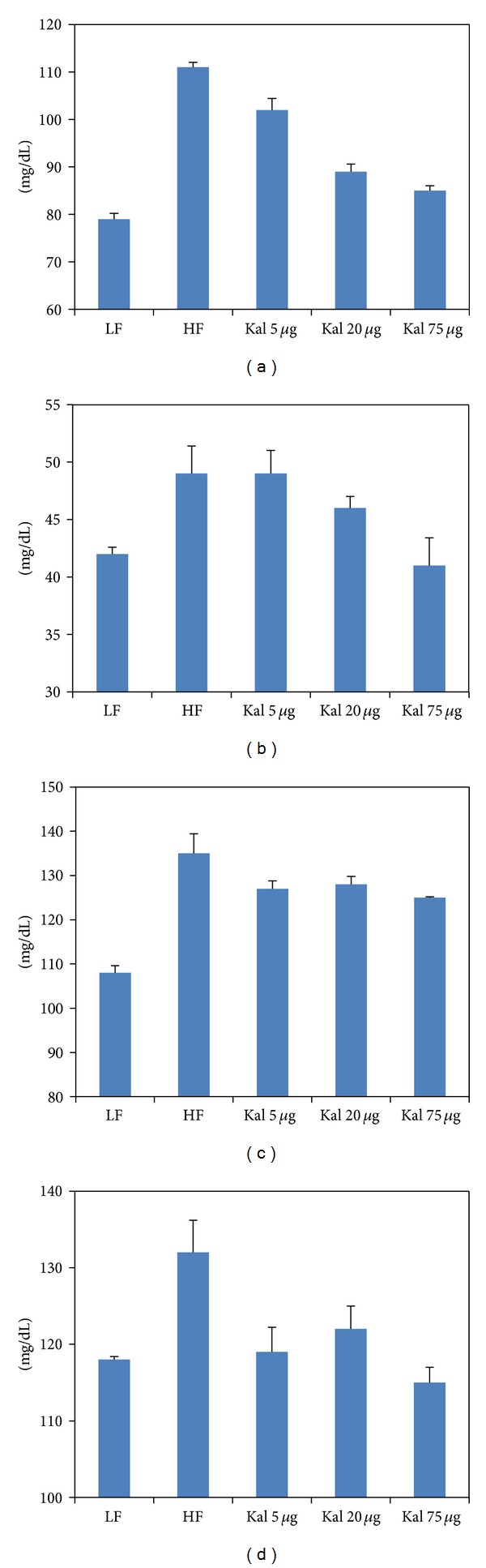
Kal-1 treats the irregularities in blood biochemical parameters in mice due to feeding on high-fat high-sugar diets at week 30. Effect of Kal-1 treatment on different biochemical parameters, namely, (a) HDL, (b) LDL, (c) cholesterol, and (d) triglycerides in high-fat high-sugar diet fed mice at week 30. Treatment with all doses (5, 20, and 75 *μ*L) of Kal-1 was started only after 21 weeks (induction period) along with high-fat high-sugar diet. LF: low-fat control, HF: high-fat high-sugar control. All the values represent mean ± SEM from five animals.

**Figure 7:**
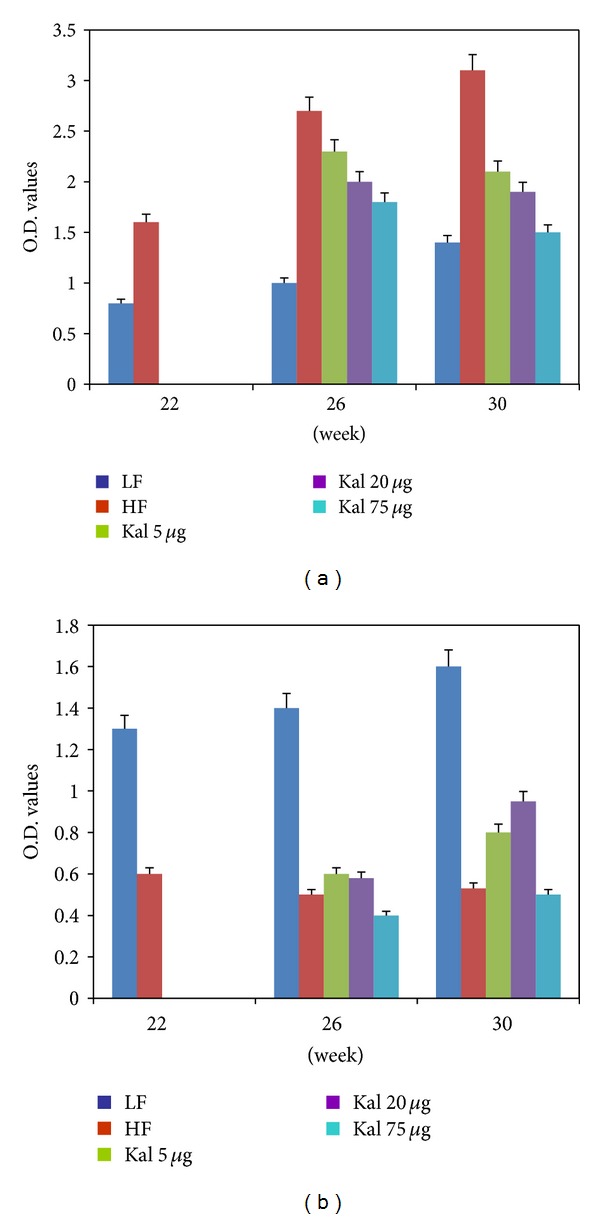
Treatment of Kal-1 brings back the levels of pro- and antihormonal levels to normal which were showing metabolic disturbed contour in high-fat high-sugar fed mice at weeks 22, 26, and 30. (a) Effect of Kal-1 treatment on leptin profile in high-fat high-sugar diet fed mice at weeks 22, 26, and 30. (b) Effect of Kal-1 treatment on high-molecular-weight adiponectin profile in high-fat high-sugar diet fed mice at weeks 22, 26, and 30. Treatment with all doses (5, 20, and 75 *μ*L) of Kal-1 was started only after 21 weeks (induction period) along with high-fat high-sugar diet. LF: low-fat control, HF: high-fat high-sugar control. All the values represent mean ± SEM from five animals.

**Figure 8:**
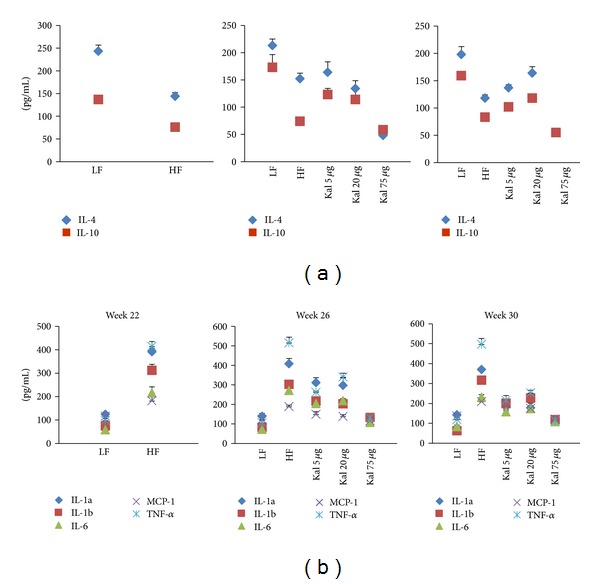
Treatment of Kal-1 modulates the anti- (a) and proinfammatory (b) cytokines levels to normal which were showing abnormal pattern in high-fat high-sugar fed mice at weeks 22, 26, and 30. Treatment with all doses (5, 20, and 75 *μ*L) of Kal-1 was started only after 21 weeks (induction period) along with high-fat high-sugar diet. LF: low-fat control, HF: high-fat high sugar control. All the values represent mean ± SEM from five animals.

In the rescue experiment, HFHSD supplemented with 20 *μ*L of Kal-1 suppresses obesity and related pro-inflammatory responses like insulin response and blood glucose levels by reducing levels of IL-1*α*, IL-1*β*, IL-6, TNF-*α*, and MCP-1 and simultaneously elevating levels of anti-inflammatory IL-4 and IL-10 at week 15; however, here Kal-1 working amount was lower, that is, 5 *μ*L (Figures 4(a) and 4(b)). On the other hand in treatment experiment, same types of profile were observed for both pro- and anti inflammatory adipokines. The only difference was in the amount of Kal-1; here, it was 75 *μ*L as observed in other biochemical parameters in treatment study. The possible explanation for this higher amount of Kal-1 is that herbal formulation with several ingredients like Kal-1 is required in little amount if this is administered with disease progression. Once disease is old and induced for a certain period of time, more than 2-fold of herbal formulation is required. To the best of our knowledge, this is the first study in which these two aspects of disease are covered simultaneously, and single herbal formulation, Kal-1, controls chronic low-grade inflammation and maintains a balance between pro- and anti-inflammatory cytokines too by its immunoregulatory effect, and then contributes to control weight gain and related metabolic problems. 

## 5. Conclusion

In conclusion, our investigations imply that Kal-1 exhibits substantial antiobesity concomitant with metabolic regulatory effect especially in terms of chronic low-grade inflammation, energy equilibrium, and linked significant disorders. However, as discussed above that Kal-1 is constituted of seven different herbal ingredients, it is very difficult to conclude that a combination or a single ingredient is responsible for these observed responses at this stage. Indeed, clinical trials are needed in order to understand the relevance of formulation. It is also clear that diet rich in carbohydrate with increased sugar may affect body weight, blood biochemistry including blood glucose and serum insulin, the levels of inflammatory markers both pro and anti, and most importantly energy balance. It would also be interesting to investigate Kal-1 mechanism and its influence on metabolic process and pathways at transcriptional level.

## Supplementary Material

Figure S1. Dose dependent effect of Kal-1 on mean body weights of mice fed on HFHSD at week 21. All doses (0.04 - 300 *μ*L) of Kal-1 were supplemented along with HFHSD. Here, the abbreviations mean: LF: Low fat control, HF: High fat high sugar control. All the values represent mean ± SEM from five animals.
Figure S2. Comparison of LF control and HFHSD control with their respective gavage control groups at week 21. Here, the abbreviations mean: LF: Low fat control, HF: High fat high sugar control. All the values represent mean ± SEM from five animals.
Figure S3. Feed consumption in pair feeding experiment a) at week 12 b) at week 21. Amount of Kal-1 was 2 *μ*L/gm body weight of mice. Here, the abbreviations mean: LF: Low fat control, HF: High fat high sugar control, PF: Pair-fed. All the values represent mean ± SEM from five animals.
Figure S4. Body weights in pair feeding experiment from week 15, 17, 19 and 21. Amount of Kal-1 was 2 *μ*L/gm body weight of mice. Here, the abbreviations mean: LF: Low fat control, HF: High fat high sugar control, PF: Pair-fed. All the values represent mean ± SEM from five animals.
Figure S5. Rectal temperature profile from week 15, 17, 19 and 21. Amount of Kal-1 was 2 *μ*L/gm body weight of mice. Here, the abbreviations mean: LF: Low fat control, HF: High fat high sugar control, PF: Pair-fed. All the values represent mean ± SEM from five animals.
Figure S6. Week wise effect of Kal-1 on fasting blood glucose in high fat high sugar fed mice. All doses ranging from 0.04 *μ*L to 75 *μ*L of Kal-1 were supplemented along with HFHSD. Here, the abbreviations mean: LF: Low fat control, HF: High fat high sugar control. All the values represent mean ± SEM from five animals.
Figure S7. Effect of Kal-1 treatment on different biochemical parameters viz. a) HDL b) LDL c) Cholesterol d) Triglycerides in high fat high sugar diet fed mice at week 21 and 26. Treatment with all doses (5, 20 and 75 *μ*L) of Kal-1 was started only after 21 weeks (induction period) along with HFHSD. Here, the abbreviations mean: LF: Low fat control, HF: High fat high sugar control. All the values represent mean ± SEM from five animals.
Figure S8. Effect of Kal-1 treatment on fasting insulin levels in HFHSD fed mice a) at week 21 b) at week 26. Treatment with all doses (5, 20 and 75 *μ*L) of Kal-1 was started only after 21 week (induction period) along with high fat high sugar diet. Here, the abbreviations mean: LF: Low fat control, HF: High fat high sugar control. All the values represent mean ± SEM from five animals.
Figure S9. Preparation procedure of Kal-1
Table S1. Composition of Kal-1
