# Supplementary figures and images for: Immunomodulatory Role of an Ayurvedic Formulation on Imbalanced Immunometabolics during Inflammatory Responses of Obesity and Prediabetic Disease
Source: Evid Based Complement Alternat Med. 2013 Nov 3;2013:795072. doi: 10.1155/2013/795072 (PMC3835817; doi:10.1155/2013/795072)

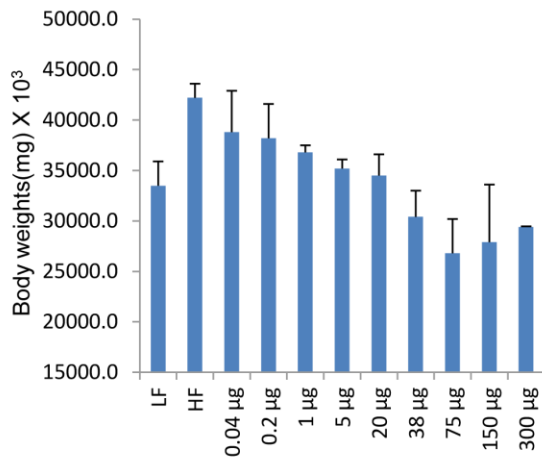

**Figure S1**

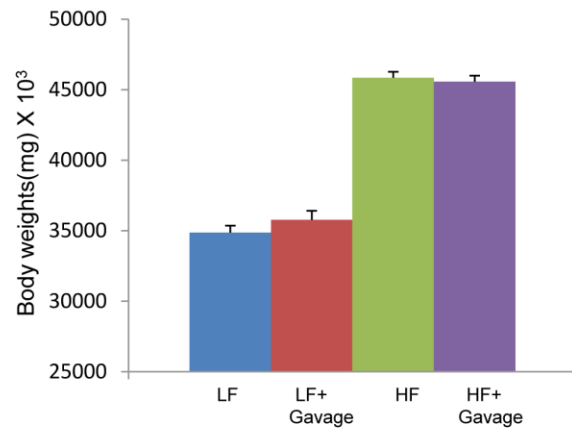

**Figure S2**

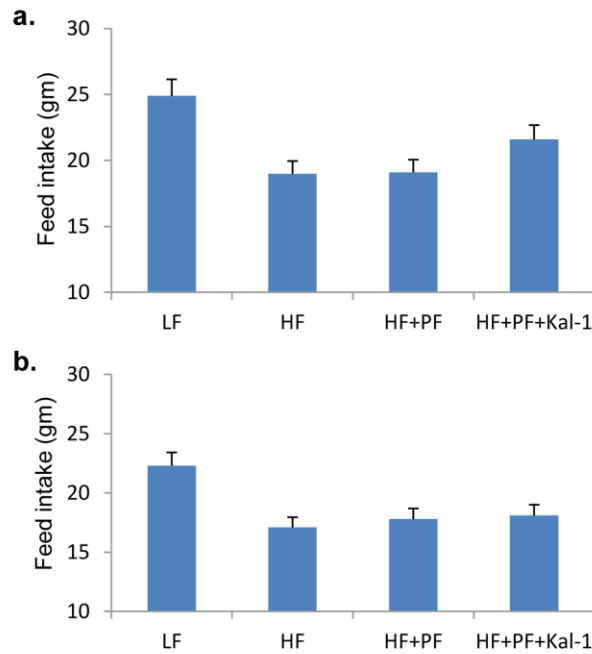

**Figure S3**

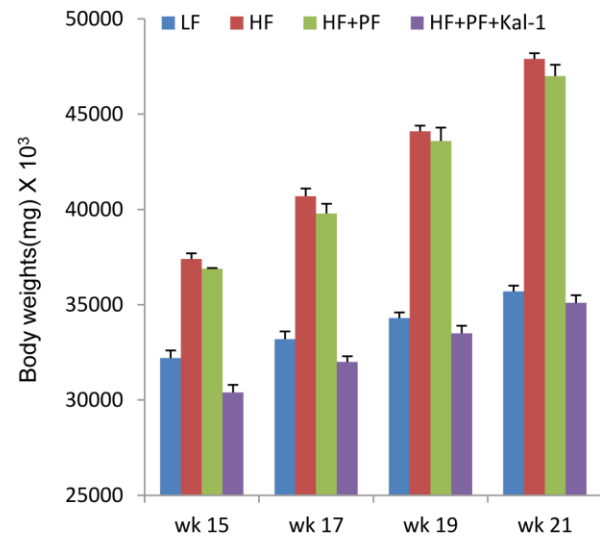

**Figure S4**

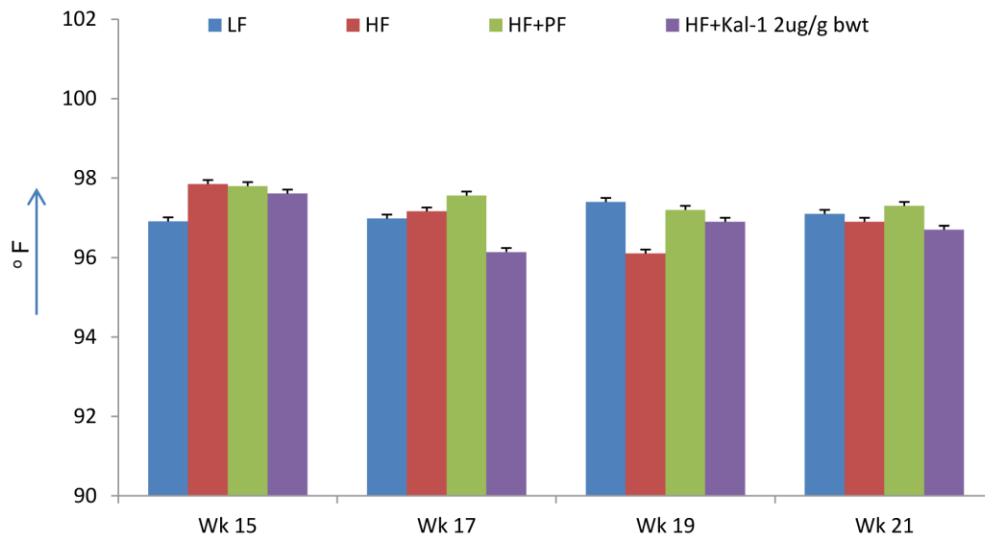

**Figure S5**

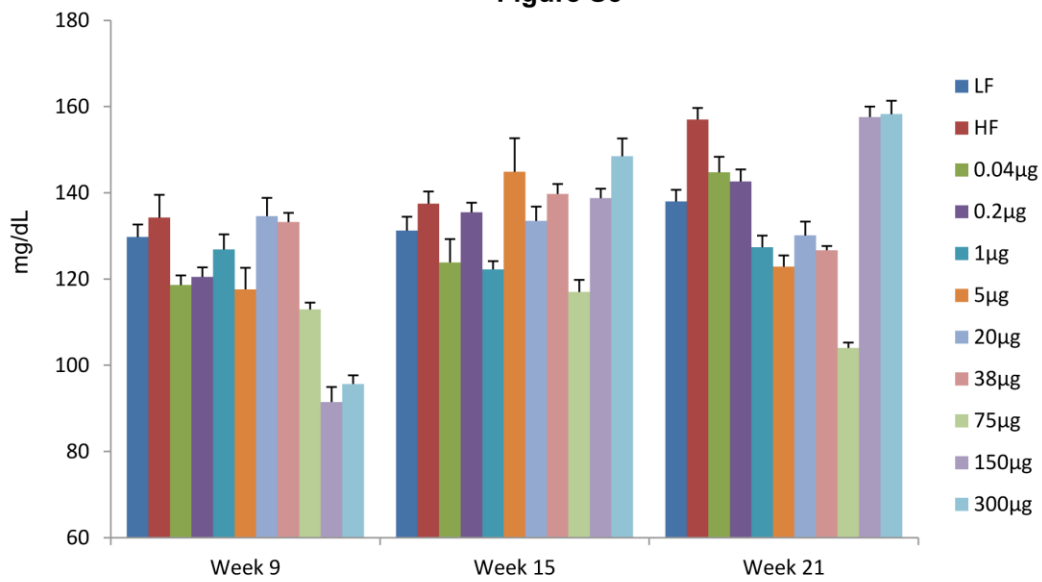

**Figure S6**

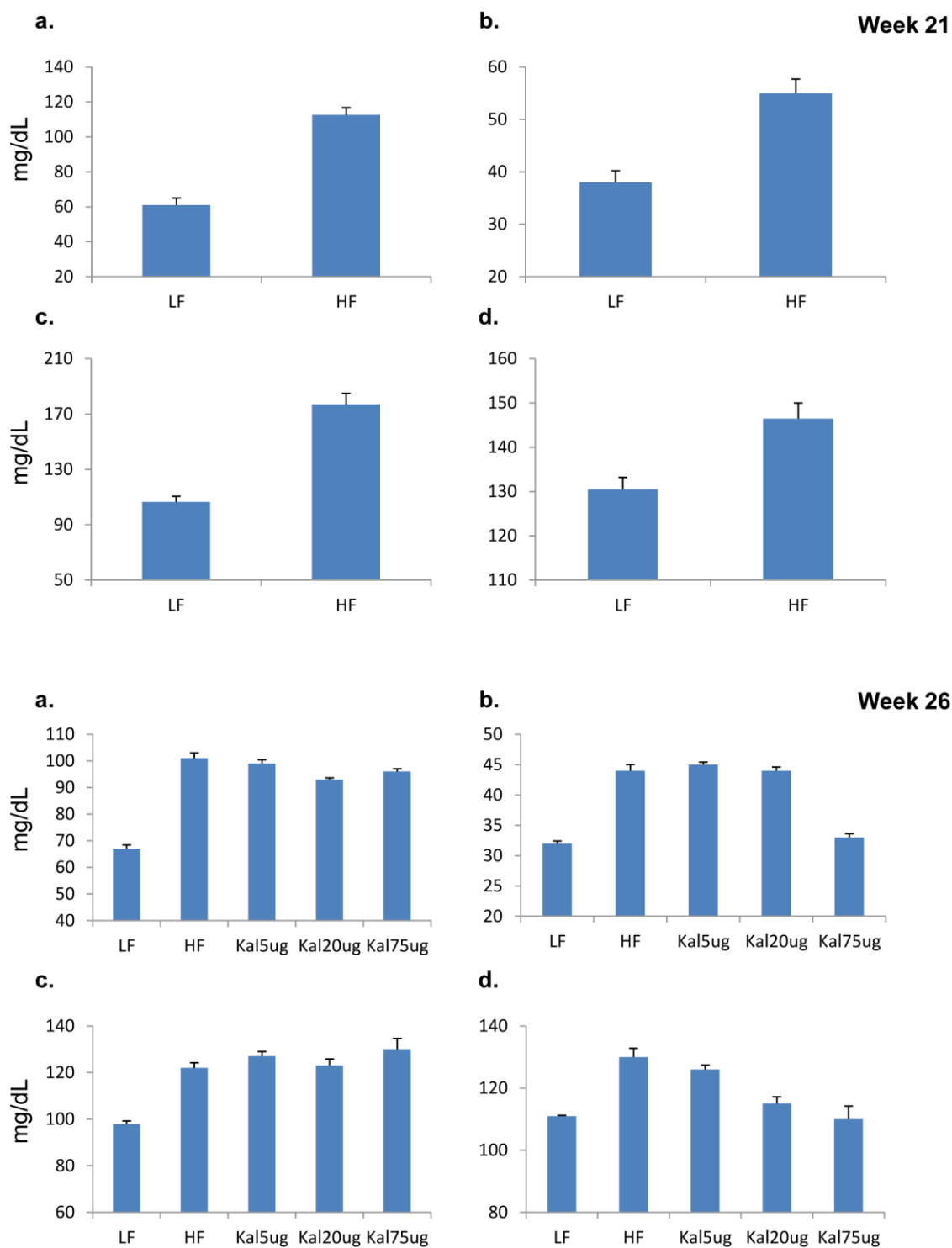

**Figure S7**

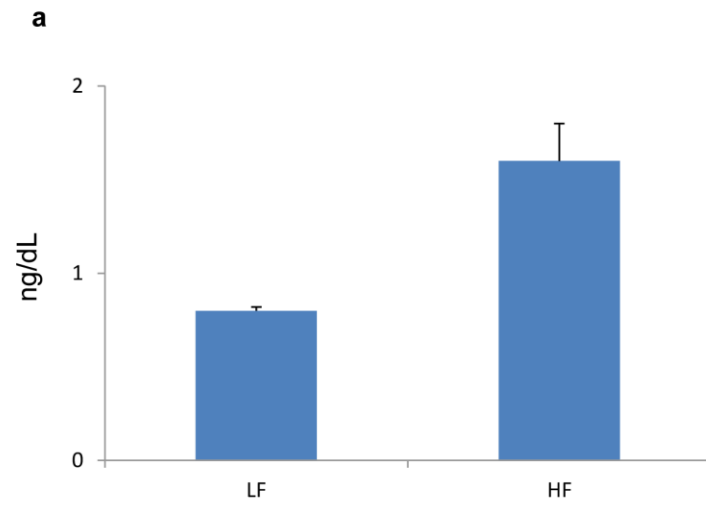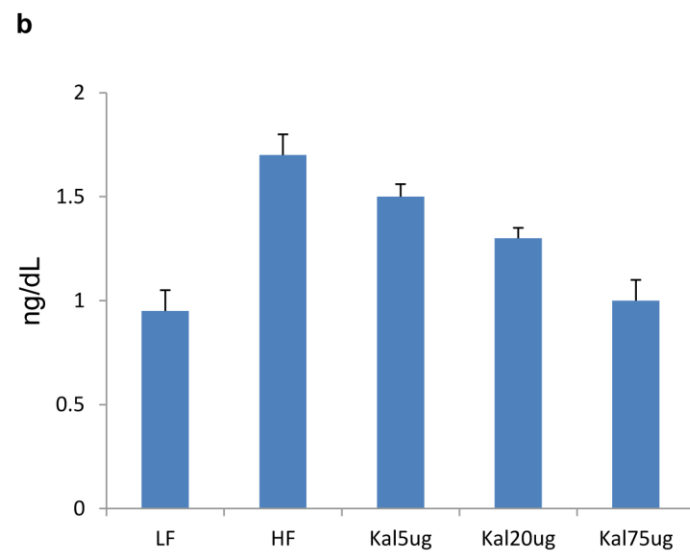

**Figure S8**

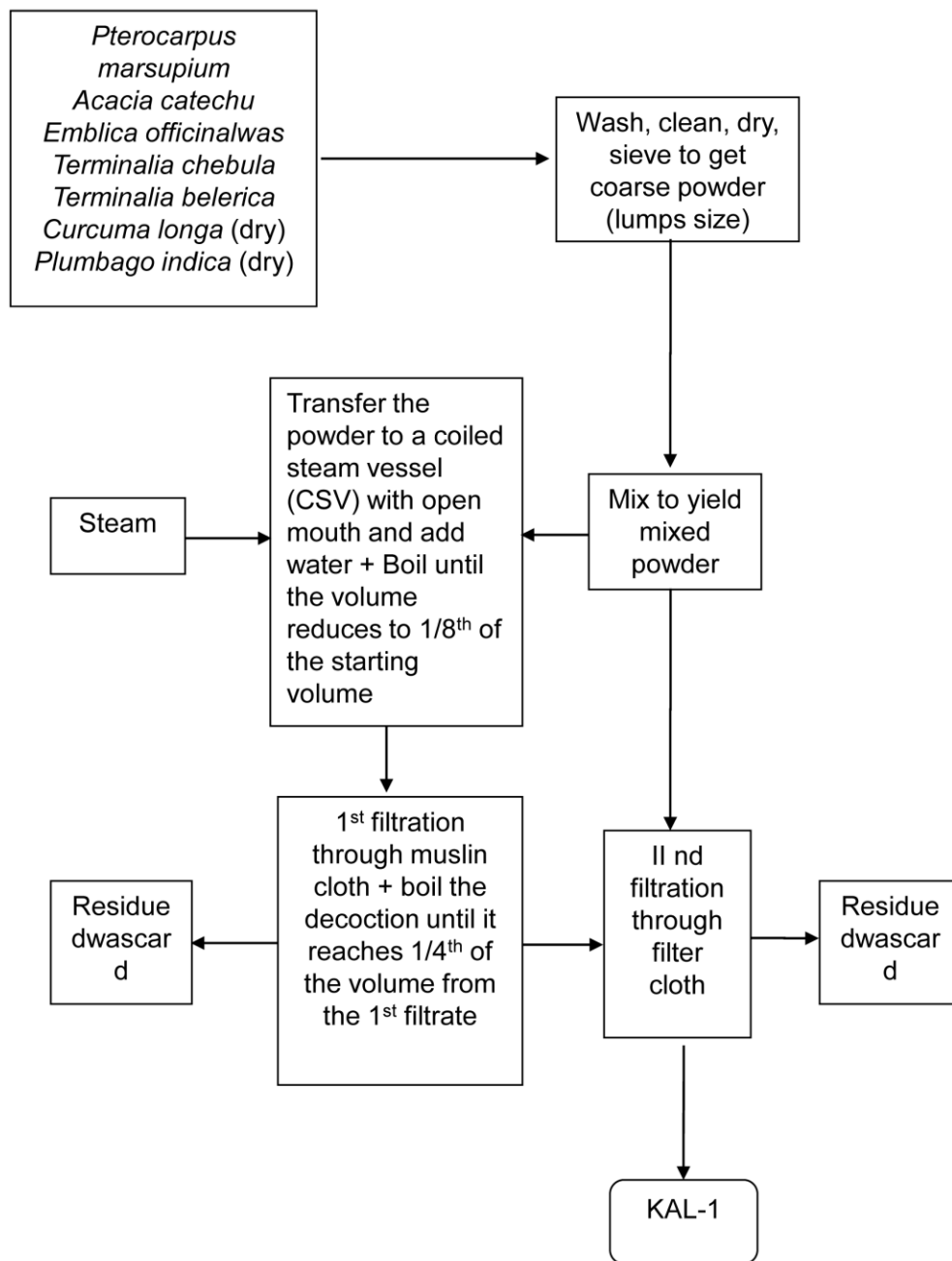

**Figure S9**

Supplement: Supplementary file 1 — Figure S1. Dose dependent effect of Kal-1 on mean body weights of mice fed on HFHSD at week 21. All doses (0.04 - 300 μL) of Kal-1 were supplemented along with HFHSD. Here, the abbreviations mean: LF: Low fat control, HF: High fat high sugar control. All the values represent mean ± SEM from five animals. Figure S2. Comparison of LF control and HFHSD control with their respective gavage control groups at week 21. Here, the abbreviations mean: LF: Low fat control, HF: High fat high sugar control. All the values represent mean ± SEM from five animals. Figure S3. Feed consumption in pair feeding experiment a) at week 12 b) at week 21. Amount of Kal-1 was 2 μL/gm body weight of mice. Here, the abbreviations mean: LF: Low fat control, HF: High fat high sugar control, PF: Pair-fed. All the values represent mean ± SEM from five animals. Figure S4. Body weights in pair feeding experiment from week 15, 17, 19 and 21. Amount of Kal-1 was 2 μL/gm body weight of mice. Here, the abbreviations mean: LF: Low fat control, HF: High fat high sugar control, PF: Pair-fed. All the values represent mean ± SEM from five animals. Figure S5. Rectal temperature profile from week 15, 17, 19 and 21. Amount of Kal-1 was 2 μL/gm body weight of mice. Here, the abbreviations mean: LF: Low fat control, HF: High fat high sugar control, PF: Pair-fed. All the values represent mean ± SEM from five animals. Figure S6. Week wise effect of Kal-1 on fasting blood glucose in high fat high sugar fed mice. All doses ranging from 0.04 μL to 75 μL of Kal-1 were supplemented along with HFHSD. Here, the abbreviations mean: LF: Low fat control, HF: High fat high sugar control. All the values represent mean ± SEM from five animals. Figure S7. Effect of Kal-1 treatment on different biochemical parameters viz. a) HDL b) LDL c) Cholesterol d) Triglycerides in high fat high sugar diet fed mice at week 21 and 26. Treatment with all doses (5, 20 and 75 μL) of Kal-1 was started only after 21 weeks (induction period) along [file 795072.f1.pdf]
