# Supplementary material for: Immunomodulatory Role of an Ayurvedic Formulation on Imbalanced Immunometabolics during Inflammatory Responses of Obesity and Prediabetic Disease
Source: Evid Based Complement Alternat Med. 2013 Nov 3;2013:795072. doi: 10.1155/2013/795072 (PMC3835817; doi:10.1155/2013/795072)
Supplement: Supplementary file 2 [file 795072.f2.ppt]

## Slide 1
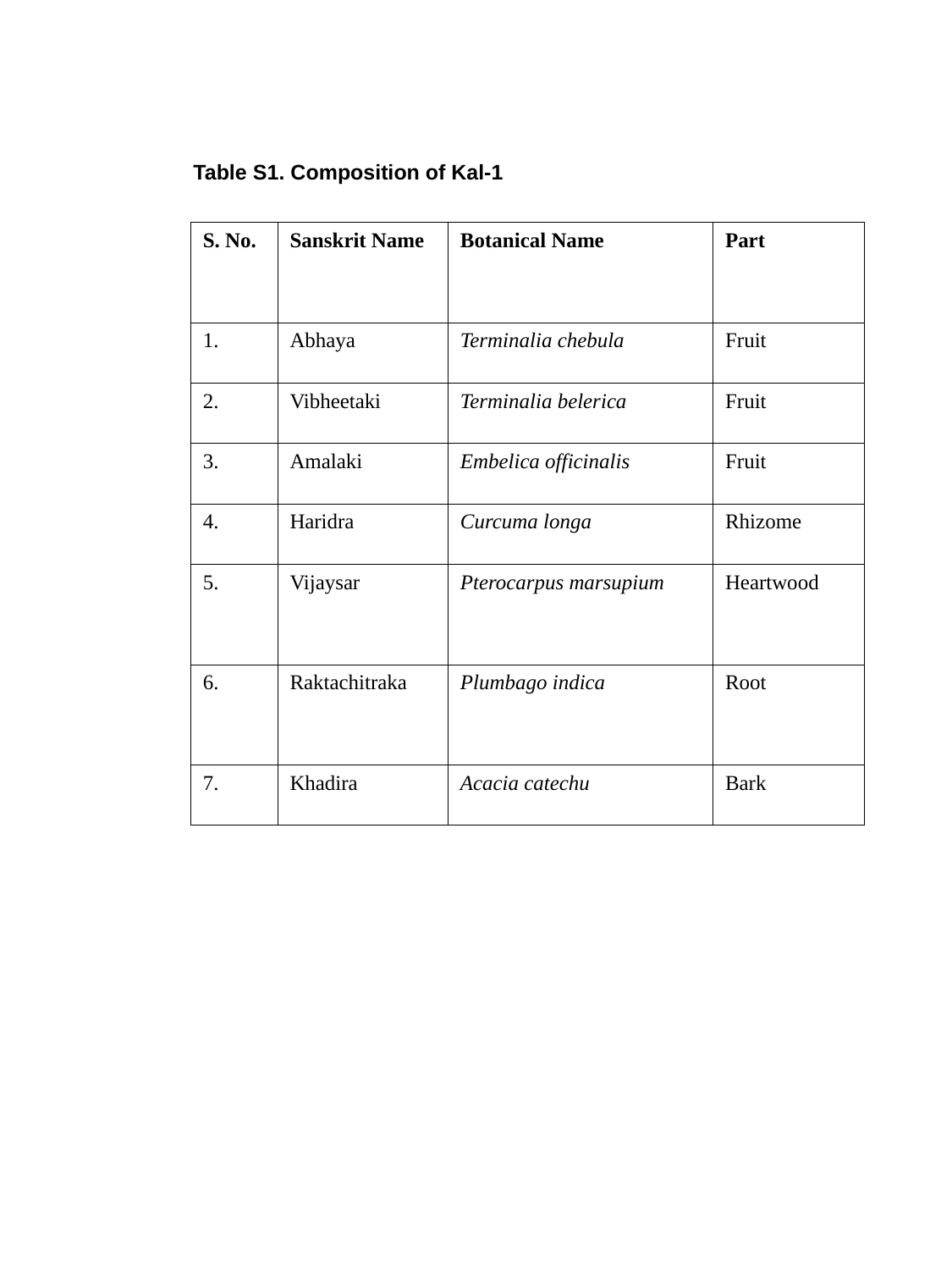

Table S1. Composition of Kal-1
| S. No. | Sanskrit Name | Botanical Name | Part |
| --- | --- | --- | --- |
| 1. | Abhaya | Terminalia chebula | Fruit |
| 2. | Vibheetaki | Terminalia belerica | Fruit |
| 3. | Amalaki | Embelica officinalis | Fruit |
| 4. | Haridra | Curcuma longa | Rhizome |
| 5. | Vijaysar | Pterocarpus marsupium | Heartwood |
| 6. | Raktachitraka | Plumbago indica | Root |
| 7. | Khadira | Acacia catechu | Bark |
